# Supplementary material for: Tunable protein synthesis by transcript isoforms in human cells
Source: eLife. 2016 Jan 6;5:e10921. doi: 10.7554/eLife.10921 (PMC4764583; doi:10.7554/eLife.10921)
Supplement: Supplementary file 1. — DOI: http://dx.doi.org/10.7554/eLife.10921.022 [file elife-10921-supp1.docx]

**Supplemental Methods**

**Alternative UTR sequences**

The natural sequences for all 5’ and 3’ UTR sequences appended to *Renilla* luciferase are shown. Sequences were retrieved from Ensembl release 75 (GRCh37). Cloned sequences begin with an additional “GG” for 5’ leaders and end with CTGCAG-polyA_60_ for 3’ UTRs.

*EEF1B2-001 5’ leader (ENST00000236957)*

CGCAGTTCCGCCGGAAGTGGCCCCAGCCTCGAGGCCGGGCGTCTTCGGTCATCTCCGGCGCTTCTAGGGCTGGTTCCCGTCATCTTCGGGAGCCGTGGAGCTCTCGGATACAGCCGACACC

*EEF1B2-201 5’ leader (ENST00000392221)*

GCCGGAAGTGGCCCCAGCCTCGAGGCCGGGCGTCTTCGGTCATCTCCGGCGCTTCTAGGGCTGGTTCCCGTCATCTTCGGGAGCCGTGGAGCGTGGGGCGCCCACAATTTGCGCGCTCTCTTTCTGCTGCTCCCCAGCTCTCGGATACAGCCGACACC

*EEF1B2-003 5’ leader (ENST00000392222)*

CCCCAGCCTCGAGGCCGGGCGTCTTCGGTCATCTCCGGCGCTTCTAGGGCTGGTTCCCGTCATCTTCGGGAGCCGTGGAGGTACGAACTTAAGACATGCCTATTTTATTAATTTACTTCCAAACGCAACGAAAGGTCCATGGACAATTTGTGGGCCATTTAATTCAGGGCCCCCAATTCGTACGTGGAGAAGTGGGAATGCAAAAGTACTTTGACCTTTAACCTTCGGTCCGGCGCGGTGGAGGGAAACGCCTCCGTCTCTATATAAGGAATTTTCCGGTCTCTTCGGGTCCTTTTTCCTCTCTTCAGCGTGGGGCGCCCACAATTTGCGCGCTCTCTTTCTGCTGCTCCCCAGCTCTCGGATACAGCCGACACC

*NAE1-001 5’ leader (ENST00000290810)*

GGTAGCGGCGAAGGGGCGCTCAGTGCGCCTGCGCGCTTGTGGAGCTGGTGGCGGCGCTCCGCAGGGGCTCGGCTGTTTTCCGCGCGGCAGGCGCGGCC

*NAE1-005 5’ leader (ENST00000379463)*

AGTGCGCCTGCGCGCTTGTGGAGCTGGTGGCGGCGCTCCGCAGGGGCTCGGCTGTTTTCCGCGCGGCAGGCGCGGCCATGGCGCAGCTGGGAAAGCTGCTCAAGGAGCAGAAGTACGACCGGCAGCTGAGGCGATTCCTCCATACATTTGACAGCTGTCTGGGCCCTTATGGAGCATACAGTGGAGTGGAAAG

*RICTOR-002 5’ leader (ENST00000296782)*

GTTGTGACTGAAACCCGTCAAT

*RICTOR-001 5’ leader (ENST00000357387)*

GTTTCCGGTGTTGTGACTGAAACCCGTCAAT

*SRSF5-005 5’ leader (ENST00000394366)*

GACGTAGGACGCGCCCTCCATTTTGTGGAGCGCCAGAGCTGCTAAGTGCGTCAGTTGTGGAGTGGCGTAGACGAGTTAAGTCCTGGTCTGCGTGGAGGTCGACGACTCCGTCGCAGACTACGGACCTGTCTGGGTCTCAGCCGCCAAAGACCCCGTCCGGTAGGTGAGTGGCTCACTTTGAGGGCAAGCCTTCTCGGATCGAGGCTTCTTCATGGCCGCTCAGATCGTGAGCGGCCGGGGCTGCTCTCTTTGCGGAGGATGGCGTCTAATGAGCGCAGTTGATTCGAGGAAGTACTAGCCGGACATC

*SRSF5-005 5’ leader (ENST00000557154)*

GCCAGAGCTGCTAAGTGCGTCAGTTGTGGAGTGGCGTAGACGAGTTAAGTCCTGGTCTGCGTGGAGGTCGACGACTCCGTCGCAGACTACGGACCTGTCTGGGTCTCAGCCGCCAAAGACCCCGTCCGGTAGGAAGTACTAGCCGGACATC

*CCNE2-201 3’ UTR (ENST00000308108)*

TAAAGAAGATAACTAAGCAAACAAGTTGGAATTCACCAAGATTGGGTAGAACTGGTATCACTGAACTACTAAAGTTTTACAGAAAGTAGTGCTGTGATTGATTGCCCTAGCCAATTCACAAGTTACACTGCCATTCTGATTTTAAAACTTACAATTGGCACTAAAGAATACATTTAATTATTTCCTATGTTAGCTGTTAAAGAAACAGCAGGACTTGTTTACAAAGATGTCTTCATTCCCAAGGTTACTGGATAGAAGCCAACCACAGTCTATACCATAGCAATGTTTTTCCTTTAATCCAGTGTTACTGTGTTTATCTTGATAAACTAGGAATTTTGTCACTGGAGTTTTGGACTGGATAAGTGCTACCTTAAAGGGTATACTAAGTGATACAGTACTTTGAATCTAGTTGTTAGATTCTCAAAATTCCTACACTCTTGACTAGTGCAATTTGGTTCTTGAAAATTAAATTTAAACTTGTTTACAAAGGTTTAGTTTTGTAATAAGGTGACTAATTTATCTATAGCTGCTATAGCAAGCTATTATAAAACTTGAATTTCTACAAATGGTGAAATTTAATGTTTTTTAAACTAGTTTATTTGCCTTGCCATAACACATTTTTTAACTAATAAGGCTTAGATGAACATGGTGTTCAACCTGTGCTCTAAACAGTGGGAGTACCAAAGAAATTATAAACAAGATAAATGCTGTGGCTCCTTCCTAACTGGGGCTTTCTTGACATGTAGGTTGCTTGGTAATAACCTTTTTGTATATCACAATTTGGGTGAAAAACTTAAGTACCCTTTCAAACTATTTATATGAGGAAGTCACTTTACTACTCTAAGATATCCCTAAGGAATTTTTTTTTTTAATTTAGTGTGACTAAGGCTTTATTTATGTTTGTGAAACTGTTAAGGTCCTTTCTAAATTCCTCCATTGTGAGATAAGGACAGTGTCAAAGTGATAAAGCTTAACACTTGACCTAAACTTCTATTTTCTTAAGGAAGAAGAGTATTAAATATATACTGACTCCTAGAAATCTATTTATTAAAAAAAGACATGAAAACTTGCTGTACATAGGCTAGCTATTTCTAAATATTTTAAATTAGCTTTTCTAAAAAAAAAATCCAGCCTCATAAAGTAGATTAGAAAACTAGATTGCTAGTTTATTTTGTTATCAGATATGTGAATCTCTTCTCCCTTTGAAGAAACTATACATTTATTGTTACGGTATGAAGTCTTCTGTATAGTTTGTTTTTAAACTAATATTTGTTTCAGTATTTTGTCTGAAAAGAAAACACCACTAATTGTGTACATATGTATTATATAAACTTAACCTTTTAATACTGTTTATTTTTAGCCCATTGTTTAAAAAATAAAAGTTAAAAAAATTTAACTGCTTAAAAGT

*CCNE2-001 3’ UTR (ENST00000520509)*

TAAAGAAGATAACTAAGCAAACAAGTTGGAATTCACCAAGATTGGGTAGAACTGGTATCACTGAACTACTAAAGTTTTACAGAAAGTAGTGCTGTGATTGATTGCCCTAGCCAATTCACAAGTTACACTGCCATTCTGATTTTAAAACTTACAATTGGCACTAAAGAATACATTTAATTATTTCCTATGTTAGCTGTTAAAGAAACAGCAGGACTTGTTTACAAAGATGTCTTCATTCCCAAGGTTACTGGATAGAAGCCAACCACAGTCTATACCATAGCAATGTTTTTCCTTTAATCCAGTGTTACTGTGTTTATCTTGATAAACTAGGAATTTTGTCACTGGAGTTTTGGACTGGATAAGTGCTACCTTAAAGGGTATACTAAGTGATACAGTACTTTGAATCTAGTTGTTAGATTCTCAAAATTCCTACACTCTTGACTAGTGCAATTTGGTTCTTGAAAATTAAATTTAAACTTGTTTACAAAGGTTTAGTTTTGTAATAAGGTGACTAATTTATCTATAGCTGCTATAGCAAGCTATTATAAAACTTGAATTTCTACAAATGGTGAAATTTAATGTTTTTTAAACTAGTTTATTTGCCTTGCCATAACACATTTTTTAACTAATAAGGCTTAGATGAACATGGTGTTCAACCTGTGCTCTAAACAGTGGGAGTACCAAAGAAATTATAAACAAGATAAATGCTGTGGCTCCTTCCTAACTGGGGCTTTCTTGACATGTAGGTTGCTTGGTAATAACCTTTTTGTATATCACAATTTGGGTGAAAAACTTAAGTACCCTTTCAAACTATTTATATGAGGAAGTCACTTTACTACTCTAAGATATCCCTAAGGAATTTTTTTTTTTAATTTAGTGTGACTAAGGCTTTATTTATGTTTGTGAAACTGTTAAGGTCCTTTCTAAATTCCTCCATTGTGAGATAAGGACAGTGTCAAAGTGATAAAGCTTAACACTTGACCTAAACTTCTATTTTCTTAAGGAAGAAGAGTATTAAATATATACTGACTCCTAGAAATCTATTTATTAAAAAAAGACATGAAAACTTGCTGTACATAGGCTAGCTATTTCTAAATATTTTAAATTAGCTTTTCTAAAAAAAAAATCCAGCCTCATAAAGTAGATTAGAAAACTAGATTGCTAGTTTATTTTGTTATCAGATATGTGAATCTCTTCTCCCTTTGAAGAAACTATACATTTATTGTTACGGTATGAAGTCTTCTGTATAGTTTGTTTTTAAACTAATATTTGTTTCAGTATTTTGTCTGAAAAGAAAACACCACTAATTGTGTACATATGTATTATATAAACTTAACCTTTTAATACTGTTTATTTTTAGCCCATTGTTTAAAAAATAAAAGTTAAAAAAATTTAACTGCTTAAAAGTAAAGTTTTGCCATTGCTTGGAGAAACTTTTTTTTCCTTCTCTGCGCTGCCAGCTGTAACACTTCTTCTGGATTGCTTGCATTCAACTCTGTCTGGCCGATGGCTTTGATCTTCCAAAACAGAAAAGTGATGTTATTAGAGGTGTGTCAAAAATTAAGTTTTGTTGGTACAAGTAATATAAAGCTACCTACGTGCTAACAACGATACAGTTTAATGATTAACTGAACCTCTTAACTGTAAAACCCAGGAGTCTTGGAAAAAAATTAACATAAAGATTAACCAGGGCCACTCTCAAGGAAAGATGGACTGCTGAGCCATAGTTTATGGAATTACTTAAGTGGCATTTTAATATATTAAGTAAATTCATGTAAATTTATTTGAAAGTATAAGTAAGCTCTACATGGCGATTTTTAGAGTTAAACTAGGGCTCCACTTGTTAATGTGCAAAAATAACTGGT

*NAB1-003 3’ UTR (ENST00000409581)*

TAGCTGTGATTTCTCTCACCGTTCTCTGGAAATGGCATCAGATTTAAGGATAATACTCCATCATAGAAATAAGCCTTAATAACCAGTGTTGCCTCATTCAGCTCAAACAGATTTCATAGCCAAAGCAAAAGGACTGGTACGGTAGTCTGTGGAAACCAGGAAGATAAAACAACAGCCACAAAAGAGAAAATCAAGAGTGTTGCAATCTATAACAGTAATATTGATTCATTCACATTCCTGTGTTAAGTCATTTTATATGGAAAGGCTTACAAATCAATATTGTAAGCATTCATTATTTAAGAATGTACAATGTATTTGTGTAATTTATAGAAGTAAAATCTAGATGTTGAGACCTGTTTGGTCTAATAGATGTGGATACAGTTTATTTTACTTGAAATTTTGTTGTCTACTTTGTGTGTTTAACGTAAATATATGTCAGAGTTTAGAATCTGCCTGCAGTTGTGAAAAAGAAAGCTTAAGTGATGCAGTTATTGGCAAGATTGCAATGATTATGGAAAAATAGAAAGCGAATACTCAGTTTAAGCCAAGGAAAATATTGTGGATTTAATATTTGATAAAACTGATTTTG

*NAB1-001 3’ UTR (ENST00000337386)*

TAGCTGTGATTTCTCTCACCGTTCTCTGGAAATGGCATCAGATTTAAGGATAATACTCCATCATAGAAATAAGCCTTAATAACCAGTGTTGCCTCATTCAGCTCAAACAGATTTCATAGCCAAAGCAAAAGGACTGGTACGGTAGTCTGTGGAAACCAGGAAGATAAAACAACAGCCACAAAAGAGAAAATCAAGAGTGTTGCAATCTATAACAGTAATATTGATTCATTCACATTCCTGTGTTAAGTCATTTTATATGGAAAGGCTTACAAATCAATATTGTAAGCATTCATTATTTAAGAATGTACAATGTATTTGTGTAATTTATAGAAGTAAAATCTAGATGTTGAGACCTGTTTGGTCTAATAGATGTGGATACAGTTTATTTTACTTGAAATTTTGTTGTCTACTTTGTGTGTTTAACGTAAATATATGTCAGAGTTTAGAATCTGCCTGCAGTTGTGAAAAAGAAAGCTTAAGTGATGCAGTTATTGGCAAGATTGCAATGATTATGGAAAAATAGAAAGCGAATACTCAGTTTAAGCCAAGGAAAATATTGTGGATTTAATATTTGATAAAACTGATTTTGTTTAACAGGAAATTTTTAGCATTCAGTCATATAACATCTGGTTATCAATGCACGTTTACACAATAAATACTTGAGTGGAGGAAAGTTAAAAAGATGAGCAATAGAGTAGAAAATATATCTTAAACTAGTTGACCTAGATTGTATTAATAGCTACTTAAGATGTTTCAAAGATAGGAAGCTATTGCTTGGACAGAGAACTTGAAATAAGTGGACCCATGTATAAAAGCTTTGACTTAAACATTGATATTTCAGAATGTGTTAAATAGATTAAGACACAGTAAGTTAACCCTACATGTTATAAAGATGGCGACTGTTAACAAAGGCTGTAACAGATTAAGTACTATTTTATATCCAGAAAGTCTTCTCTATGTAGAGAAGTCAGAGAGACTAGATGCTTTCACTAGGGAATGTCTTCCCACCCAGCCATCACAAATGTGGACAATCACTGCATCCACATCTGTAGGCATATTTCTATGGAAGTTTAATTGACAGCTATATTCATTATTTATTTTACAATTTCATTTTTCTACACCTTTGAGATTTATGAATGCAGTTTTTTCTTAAAATTTATTTTAACTTGACAGTATGTTTTTAGTTCCCCCAATTTAATTAATGGACCATGTGCATATATATGGGAGTGTGCTTACATGTTAATAATTTACTTGCATACTTATGAGAATTTCACATTGGAATTCATAATGGTAAAACAACATACATCTGCCAATATACGTTTTTTCTGTTGGTTTAAGAGAAGATAACTGACAGCTTTACCTACTTCCTACAGATGCATCTAAACCCAGATATTACTGAGAAGAGTGTATTGACTCTGAGTGTAAGAGAGTATGTGTTTTTTTGTTTTTAGTTCTGCTCTAGATCATAATTGTAAAAAATATTAAGTCATAATCTGTTACACTAAAATTTGTCAGCCAAATGTTAGATGAAATGTCTGCACTGTAGTCTCAGATCACTGTCACGTATATAAATTGCTTCTTCATTTTAATTTGTAGAAGTACTTTACAGTAGGAAACGCCAGTAAACAACTTTTATACTGTTAAAAGGCTTTTTTCCCCTTCCTAAATGTTTTAATTGTACCATAGTGTTTTGCTCACTGAAGAAGCTTCTTATGGACCTTGCAACTTTGTTGCTAGCTTGAGGTTGATTATTGTGGTTGTATTGTTCACTGTGTGTAGAAATAGTATGAGTACGATTTCAATAGACTGTTCAGTTTTTAATATTAGCCATAGCACTGGTTAGTATATCTCAGTAGTTTCATGAAACGTTTCCTGTATTCTAATCTATTTTGAAACATTTTGTTTTTTTTTAATTGTGTCTTACAGTCAAGTTTGTAGATTTTCATAAGCCACAATTTTAAAAGATGCAGTAATCTTCCAACTTCCAATATTTATCCATTCGTTGTGGACCCACAGATTGCATCTTTAAATTCATAATAAGTTTCCTTAACTATCTTATGTTTCTAGTCTTTCAAGCTTAGTGATAAGGTGGAAGCACAAGAAAAATTTCAGTAGAATACAGTTTTTATTTTGTAAACACTAATGTATTAAACTTGCTATACATTAAAGCAAATAATATATATTTTTATTTGAATTGTATATGTGAATTGGAAGTTATAATTAGTTGATTTTTTCATTTTGTTAGAGGTATTTTCACTGAACAAGGTCAATTGGTTACCTCAGTATTACAGCCAATATAGTCCAAGGGACCATTTCTCCCCGAGTCTCTTACACTTTATTGTGCGATGTCCACGTTTTTGTGACTCTTCAAGCTGTTGGTGAGGTGGGACGAATGCACTTGCTTCCTGTGGCAATAAAGATTTTCTGTGCCTCACA

*NDC1-201 3’ UTR (ENST00000234725)*

TAGTTAAGTAATATAAACTGTGTTCATTACACTGCTGATACAACTACAGATGGGACAGTAAATGTTCAGCATTCTTGGATCAG

*NDC1-001 3’ UTR (ENST00000371429)*

TAGTTAAGTAATATAAACTGTGTTCATTACACTGCTGATACAACTACAGATGGGACAGTAAATGTTCAGCATTCTTGGATCAGAAGAAAACGGACTAATTAGATGCTTCCTTTGTCGTGGTGGTTGCTTTGAAAACTATACTTTAATGGGAGAAATCATGGAAAGAAATTCTCAACAGAATAACTGAAAACTGCCTTTTCTGTACCGATTGCTTTTTGTGTGTGTGGTATAATAAAATCTTTATTCAATTTTACAGAAGCATTGATGGCAGTCGAAATGTCTCTAGCTCATATAACTTAATAGTAATAACTAAAAAACTTTTAGAATTTACTTTTGAAAGGAGGGAAGCCAGTTCTGAAATGAGTATAGGTTGATTTCATAGTCTTCTTAATTAAGAGTTTAGCTCTTTGTAAACTCAAAATACATAAACTTTTTAAGTGTAGTTTCATTTACTGAAGGATAAAAATGGTAACAGTGCAGCAATATTCACAAAAAATATTGTCTAACGGACATATTTTGTTAATCTGTTAGGTTGGGTTTTTGTTTCCAGGGACAAATTAAATTTGTATGATTACCCAAAAAAGGGTCTCAGTTTACAGATGCTAACTCTATATAAAGGAATGTGGAAAAACTCAGTTCTTAAGTTACAAGATTAAAAATTCACATTTGGTCTTTAAGAAACAATTGACTGACATCTATGAATTTATTTTGTATCATGCTAGTAAACACGAAGTATTAATGTATGGGTATTTTCCCAGCTAGTTTTGCTTTCTTTTTCTGGAGCAAAACATTAAGTGATTGCAGAGTTTTTCAAGCAAGAGAAAAAGGTTTGCAAAAAAACCCAGGAAATGTTCCCTTTTTTCCCCACCATTCATCTTCATTAGATCAAATTCTGTGAAACTTGTCTGGTCTCTCAAAGGGAGCAGCCTCTGTAGTGTTAAATGGCTAATTAAAATAGGAAGATCTTTATAGCCAGAAACAACTTAGTCATCAAATAGCAAGTGAAACCAAAACGTCAGAGGGATTACTGTACTTGGAAGTATGTTGTGTGTCCCAAATGTGAACGAAGTATTGTTAGAATTTATTAGATCAGCTTCTTTGGAGATCAAAGATTGGAAATCCTAGTCATAGATATTCACTGGACTGGCTTTGGACTGAAATGCTCCTTTGTAATTCTTTTCCTATTGTCTTTTCCTTCTAGTGTCCCAAAATATTTTCTTTAAAGTCAGCACAGTACTGTATATGAATCTTTAATGTGGTATCATATATGTCTACTTTTGTCTGATTCATCGATGTATTATATCTTTATAATTGAATATTTTAGCTCCGGGTCCTGTTGCCCCTTCAAGCAGTACATGCCAAATTATAAATAGGTGCTACTGGCCTTGAGCATATCACTGTGGGACAGTTCCCCAATTGTCAAGTGTTTAGATATGTAGACTATTGCCATTTGTTTTTTTGTTTTGGTTTTGCTTTGTGTCTGAAGCTGAATTGATTTCTTTTTTTTGAATGTGAAAGTTGAATTTCAAACGTAGTCATTTCTTACAGATGGCCAAGACAGAAAATTGTGGCTAGGTTGACTGAGAACTGTTGTCTTCCATGTATTAACACAATTAAGCTTTTTATATTCCACTCTCTGTGCTGACCCTGGCTGAGGCATTTTGGGAGACAAGGACTCTGAATCTTCTGCTTCCATTAAAGAAGAACTGTGATATTCAACATTGGATTTCTGAGAATAAAGATAGGATGATTCCTTTGAACTTTGACTTACTTGTATAAAATGTCCAGCTAGGTTAGGTTTTTGCCATTTCCTATATACTTTGGGTAAAGCTACATTTGATGAGCAATGTGAATGTTTCTGAGAATGTTCATTCCTGTTTTCTCTTAAGAGAATGTGCTGTGTACTAAATACAGGCCACATAGTGTCTGCCTGTTGAAGATCTGGAAACTGCCTCCCCAGATCTGTATTGTATTTGGTAGGTAAGGGGGTCAGTTTCTTTTTCTCATTGTGTGTTGATAATCTACACACCATCTGTTGGAACCAGGGTGTTATTATGGGGAACTCCTCCTGTGTACTAGGAGGAGGACCTTAGGGAGACCAAGAGGAGAGAAGCATTTCCTTTGATGAAGTCACATCCTGTCTATGAGCCCACTAATGCTGTAACATTGGCCTGAAAGAGAGTGTTCTTTAAAAGCCTTTCTCGGCTGTTAGTATAAAAACATGATGGTATCAGCTCTTAGCATGTTTGCTTGACCCTTATGGAAGGTATAAATCCACAGAACTTCCTTCCCAGAGAACTGGGAAATTGTCCTAGAAATAAACCTTGTACAGTTGAGTGGACATGGATAAGCAACAATTTGTTACTTTGCAGGATTTGTTCCTTGGTAATTGTTTGGTGTGTCATCCTGTAAATATTCATGATAGTCTGTTTATATCCTTTTGTATATCGTTGATACTGGATTGGGTAGAAAAATAAATTGGCAATTTAAAAAAATGGAACAGTTAATTGAAA

*RICTOR-002 3’ UTR (ENST00000296782)*

TGACCTCATATTTATGATGGATATAGATACATACTATATATATTCATATTTGTGGATTTCCTAAAAGCCTCAGAAAATACGACTGACTAGGCAGCAAAGACAGGAGTATCTTCTGTACACTGTTCCGCAGTTACTGGTACATGAACAGTTGGAACTGCTGACTTTCCTAACCAAAACAACTTCCTTCTCTCCTTTGTTGAGCCTTTTGAGGGGTTCATGATTCATTACCACAGTTTTAAGAGTTTCAGTTACCATTGTATGCAAGAGCCAAGCACTGAATACCTACATAGGTTTTCTATTTTCTTTCATTTTAAAAGCATAATGACAGTGGAACAATAATGGGATATGCAGAAGCACCCTTCACAAGTTATTTCTGAATGATTTTTAGGGTAAATAATACAGATGCCTTGTTTGTTAACTAACTTGTGGAAAGCAGGAATCAGTGTCTCTAAGGCTGCATCCTATTACCACAATGGGGTGTGCTATAACTGCTGGTATTAGAGAGGGAACTTTGGCCCTTTCACGTTTTTCTTAATGTTTGTAACACTACTTCAGAGGTTTATAACCTCAAAGCAGAAGAAGAGCCTCAACAACCCGGGACTTATAAGTTATTTTTATGTTACTAGACTTGCATAAAGATTCTTGTTTTCCAACTCTTCATTTTGTTGCAATGTGTTATTACAGGATATATGAACCAATTAAGGTTTTTCACTACAGTTCTTGAATAAAATTTAAAAATCATTTTTTATTTTAATTAAAAATATTTCCCATTTATAGAATGCATATATTTGCAATGGACTTCCACTTTCATCAACTTTCCATCTCATCGCTTTAAACAGGAACTTGAACAAGCACTGTTAGTTTAGACCTAAAGGATAGGAAAGCATTAAATAATACTTTGGATCTCCTGAGGAAAAGATAAGTTTGCTTGCAATTTACACATTCCATGGGGAAAGAAGAGCCATATTTCCTTAAAAAAAACATTAATAAAGCTTGTTATTGAGAAAAATTGTAGTGAAAAGCCTTAAGTACCAAATTTTAAAGCAGCAGTAACTTAATTTTTATATCAGTGTTTTTGTTTTGCACAAACTAAATGCAGTGGTAGGTGGGTTTATGAGTATATTAATTGCCTTTATCCATTTGTGAAGTTAAGTTGATGAGGGCAAGGTTTTTGTTTGTTTAATTTGTATATGTCTAAAGGTATTTGGAACTTTTTACAGGAATTAAACATATATGCAAATTTGTATATAAAAATAGCATGGCCATCATTTGAATGCTTGTAAATGAAAGGATTATCTTTTTTGAGATCTATATATAAATAGAAATAGAAAATCCAGCTGGACTGATTAGGATTCTTTTTTAATTCATTTGTGTATAACATTTTTATTACAATTACACATCAGTTTTGACACAGTCATAGCAACATTAATATTTTCCCATGATGCAGATCCTTTTTGTAATGGGCTTGTTCTTTGAGATCTCTGTAAAGAACCCTGTGAACTAGAAAACATAACTCACAGAGATACTTTTTTAAAAAATTTATTTACTGGAACTGAAAGTTCCAGTTGGGATGAAGCATTTCATCTCACTTCATAACACCTCTTTGACTGCACTTCAGTGAATTGTTCTTATGTGCACTGTGTAGCAACTTACATTATAACAAAGCAGATAAGGGCTGTAAGCTGCTGCTTATGTTGAAAAGTGGTTCTTCAGATTTTCTCTCATAAAATCCAGTTGAAGATAAATAATTTTTTTATACTTTATCACTGAACCCAAGTGTTTATTTAAATGTCAACAGTACTTCTAAGAACGTTGCCTGTCATCGTGGTCTTTGGTCTTGGATAACTAAACTGCCTTTCCAGAGAACCAAATGTCAGAGTTACTAGACCAAATAGTGGTTAAAACCTCCAAAGGAAGTAATGTAATCTTATTCATAATGGGATTAACATATTTTAGACATTCATTTTAAACACTACCTCAGTTAATATAGAGTATAAAAATCTGTGGTTTAATCCCTCAAAAGTTAACAGTAATTTTTTTTTTGTCTTACACACACACACACCCCCTCCCCCACCATCACTATCCCTGTACCCTCACCTTGGTCATCTATCCTGAAATAAGGCTTAGTTAGTATTGGCCTGAATGTTTTGTGTTTTTTTTTTTGTTTTTTTTTTTTACTGTTACTTTGAAAAATATGTATGTATACCTTATCATATCTGCCTATATCACTTACTTTGGGGAGATACTCAGAGCTTTGTGGTTATCAGTATACTAAAAAAAAAAAAAA

*RICTOR-001 3’ UTR (ENST00000357387)*

TGACCTCATATTTATGATGGATATAGATACATACTATATATATTCATATTTGTGGATTTCCTAAAAGCCTCAGAAAATACGACTGACTAGGCAGCAAAGACAGGAGTATCTTCTGTACACTGTTCCGCAGTTACTGGTACATGAACAGTTGGAACTGCTGACTTTCCTAACCAAAACAACTTCCTTCTCTCCTTTGTTGAGCCTTTTGAGGGGTTCATGATTCATTACCACAGTTTTAAGAGTTTCAGTTACCATTGTATGCAAGAGCCAAGCACTGAATACCTACATAGGTTTTCTATTTTCTTTCATTTTAAAAGCATAATGACAGTGGAACAATAATGGGATATGCAGAAGCACCCTTCACAAGTTATTTCTGAATGATTTTTAGGGTAAATAATACAGATGCCTTGTTTGTTAACTAACTTGTGGAAAGCAGGAATCAGTGTCTCTAAGGCTGCATCCTATTACCACAATGGGGTGTGCTATAACTGCTGGTATTAGAGAGGGAACTTTGGCCCTTTCACGTTTTTCTTAATGTTTGTAACACTACTTCAGAGGTTTATAACCTCAAAGCAGAAGAAGAGCCTCAACAACCCGGGACTTATAAGTTATTTTTATGTTACTAGACTTGCATAAAGATTCTTGTTTTCCAACTCTTCATTTTGTTGCAATGTGTTATTACAGGATATATGAACCAATTAAGGTTTTTCACTACAGTTCTTGAATAAAATTTAAAAATCATTTTTTATTTTAATTAAAAATATTTCCCATTTATAGAATGCATATATTTGCAATGGACTTCCACTTTCATCAACTTTCCATCTCATCGCTTTAAACAGGAACTTGAACAAGCACTGTTAGTTTAGACCTAAAGGATAGGAAAGCATTAAATAATACTTTGGATCTCCTGAGGAAAAGATAAGTTTGCTTGCAATTTACACATTCCATGGGGAAAGAAGAGCCATATTTCCTTAAAAAAAACATTAATAAAGCTTGTTATTGAGAAAAATTGTAGTGAAAAGCCTTAAGTACCAAATTTTAAAGCAGCAGTAACTTAATTTTTATATCAGTGTTTTTGTTTTGCACAAACTAAATGCAGTGGTAGGTGGGTTTATGAGTATATTAATTGCCTTTATCCATTTGTGAAGTTAAGTTGATGAGGGCAAGGTTTTTGTTTGTTTAATTTGTATATGTCTAAAGGTATTTGGAACTTTTTACAGGAATTAAACATATATGCAAATTTGTATATAAAAATAGCATGGCCATCATTTGAATGCTTGTAAATGAAAGGATTATCTTTTTTGAGATCTATATATAAATAGAAATAGAAAATCCAGCTGGACTGATTAGGATTCTTTTTTAATTCATTTGTGTATAACATTTTTATTACAATTACACATCAGTTTTGACACAGTCATAGCAACATTAATATTTTCCCATGATGCAGATCCTTTTTGTAATGGGCTTGTTCTTTGAGATCTCTGTAAAGAACCCTGTGAACTAGAAAACATAACTCACAGAGATACTTTTTTAAAAAATTTATTTACTGGAACTGAAAGTTCCAGTTGGGATGAAGCATTTCATCTCACTTCATAACACCTCTTTGACTGCACTTCAGTGAATTGTTCTTATGTGCACTGTGTAGCAACTTACATTATAACAAAGCAGATAAGGGCTGTAAGCTGCTGCTTATGTTGAAAAGTGGTTCTTCAGATTTTCTCTCATAAAATCCAGTTGAAGATAAATAATTTTTTTATACTTTATCACTGAACCCAAGTGTTTATTTAAATGTCAACAGTACTTCTAAGAACGTTGCCTGTCATCGTGGTCTTTGGTCTTGGATAACTAAACTGCCTTTCCAGAGAACCAAATGTCAGAGTTACTAGACCAAATAGTGGTTAAAACCTCCAAAGGAAGTAATGTAATCTTATTCATAATGGGATTAACATATTTTAGACATTCATTTTAAACACTACCTCAGTTAATATAGAGTATAAAAATCTGTGGTTTAATCCCTCAAAAGTTAACAGTAATTTTTTTTTTGTCTTACACACACACACACCCCCTCCCCCACCATCACTATCCCTGTACCCTCACCTTGGTCATCTATCCTGAAATAAGGCTTAGTTAGTATTGGCCTGAATGTTTTGTGTTTTTTTTTTTGTTTTTTTTTTTTACTGTTACTTTGAAAAATATGTATGTATACCTTATCATATCTGCCTATATCACTTACTTTGGGGAGATACTCAGAGCTTTGTGGTTATCAGTATACTAAAAAAAAAAAAAAGTCTACGCTTAAATTTATAGTGCTATTTGGTTTCTCCATGATTTCACTGACAGGTCTAATACATTTTCTTTGAGTACTTGTTTGTAAAAAGTAGACTTTATGGTGAAAAATACATGCAGTGCCAAGTGATTAACTTAAGTGTTTAAAAATATTAAATTATAGCAGAAGAGGTTAGGAATGATATCAGCAGTAATAGAAATAATTGAGAAAATCATCTATAAATAATAGATATTACAGACTATAGAATACCAAAATAATGTCAATACTGTAGTTTTTAAAGATTTTAGGATTAATCTTAGTCCATATAAATTTGTACTATTGGTAATTATTGAATAATTGGGAGGAATCTGGGCAGTTGTGCTGGTTGTAAACTATGAATTTCTAATCGTAAAGTGAATTGTTATTTCTAATTGAACTTTTTTTCAAGAACAGATTTCAGCCTCACATACTAAGTAAATACTGATAAATAAGGAAATTAGAAATTTAGTATTCATAATTAAATATGCTCTAAAATTTCCTATACTTTTATTTCCTGTTTATTCTTAGGTAGATTGGAAGGGGGAAACAGTCTGTTCTCCCTAATTAAATTTTTTCTAATAACGATTAGTAGAATATGGACATTCTATATGACAGTGACATTAAAAGAGGCTCTTTGGAAGTATATACATTATTAACATAATGTGTACAAGTCCTTTTGAAATGACAACTTTAATGGGTTTCAGCTCTTTTATCTAGAGCTTGAGATAATTCAAGCTGAGTTTTTCAGGGCATATCACAACGGCCAAGTGTTCAGCAGTGGGATATCAATGCTTATTTACATTTTCCTACTGCTATTTATATAAAATGTTATTCCATTCAGAGGATGCCTTTTATCCCCACATTAAAGCACAGATCATTAAGCAATAAAAACCAAATTGTCTGTCATTCAAATTATAACTGCAGTTATTTTTGCATGGTAAGAGTGAGGTGCTAATTTTGTGTGAGATGAACTTTGTAAACTACTTTGGGAAATGTTCTTTGGAAGTAAGGTTTTTTCTCCTTTAGTCTTATGCTTCCACTTTTGTCTCAGATTCACAATCCATTAAAACATGGGGAAAAAAGAAAAGGTAAAATTGAGAGACTTTTGTTAGAGGAGCTATTTGGAATGAACCAACATTTCAGATTTTCCAAAATGTAAGTTAGGAAGTCTCCATTGTCTCTGCATTAACAAAATACACTGTTACTATCTTAATCTCAAGAGTGTCATTACAGTGAGAATCTCATTTAAAAGCATACCAGTGAAATTAATAGCAGTGCTTATCAAAGAACACTGAAATCTGTGAGAATCTTTCTAGGAGCATTCTTTTCTTCTTTTAGTTCCAAGTTCCAGGGTATTTTTCATTCCTAGTAGGTTTATATGACTCACAGAATGTGGACTTTTTTCCTGTTTGGAGTATTTTTGTAATGTAAGTATCGGATAGCTGCACCACAGCATGCATAAATTGCACATTTTGTTTTACTTTCTTTATAGAATATTTAATTTCAAAAATATAATTTATGCCAAAAAAAGCATACCTTTCAATTTTGCTACTTGGTTGATTTAGCACAAAATGCAAAGTCTTGGGGCAGAGAGGGGGAGTGAAAAAAATTTTATAGGTAATTGTTACAAAAATACCTGTCAGAAACCCTAAAGCTGCATTGTAAAACAAATGGTGTAAACTAGTTTTGAAAAGTGGTAAGGAATTGTGAAAAAAATCTCAGACTTAATGCTCTCTAACCACATGAGTTTCTTCTTTTTTATTTAGTAATACGCTGCTACATATTTGGAGGTTCTGGTGTTTGTAGGTCACTGAACAGACATTGAAATCTGATTTATATTGTATAACTGTAACATAGAAAGAAAAAGTATTTATATTTTTTCTGTAAGAATATTTCATTGAGTTGTGTATAATTTAAATAAGATTTGTCCCCAAATGGTTTTGCTCACCTTGATTTTTTTTGTTGTGATTTTCTTGTTTTTGTATAATGTGTATAGTTTATGTCAAGGGCATTAAAAGCCTCCTGAAGCATAATCTTATCAAAGGGATACATTGTTAATAAAATGTACTTAAAATTCTTAAAC
